# ORCO: Ollivier-Ricci Curvature-Omics—an unsupervised method for analyzing robustness in biological systems

**DOI:** 10.1093/bioinformatics/btaf093

**Published:** 2025-02-28

**Authors:** Anish K Simhal, Corey Weistuch, Kevin Murgas, Daniel Grange, Jiening Zhu, Jung Hun Oh, Rena Elkin, Joseph O Deasy

**Affiliations:** Department of Medical Physics, Memorial Sloan Kettering Cancer Center, New York, NY 10065, United States; Department of Medical Physics, Memorial Sloan Kettering Cancer Center, New York, NY 10065, United States; Department of Biomedical Informatics, Stony Brook University, Stony Brook, NY 11794, United States; Department of Applied Mathematics & Statistics, Stony Brook University, Stony Brook, NY 11794, United States; Department of Applied Mathematics & Statistics, Stony Brook University, Stony Brook, NY 11794, United States; Department of Medical Physics, Memorial Sloan Kettering Cancer Center, New York, NY 10065, United States; Department of Medical Physics, Memorial Sloan Kettering Cancer Center, New York, NY 10065, United States; Department of Medical Physics, Memorial Sloan Kettering Cancer Center, New York, NY 10065, United States

## Abstract

**Motivation:**

Although recent advanced sequencing technologies have improved the resolution of genomic and proteomic data to better characterize molecular phenotypes, efficient computational tools to analyze and interpret large-scale omic data are still needed.

**Results:**

To address this, we have developed a network-based bioinformatic tool called Ollivier-Ricci curvature for omics (ORCO). ORCO incorporates omics data and a network describing biological relationships between the genes or proteins and computes Ollivier-Ricci curvature (ORC) values for individual interactions. ORC is an edge-based measure that assesses network robustness. It captures functional cooperation in gene signaling using a consistent information-passing measure, which can help investigators identify therapeutic targets and key regulatory modules in biological systems. ORC has identified novel insights in multiple cancer types using genomic data and in neurodevelopmental disorders using brain imaging data. This tool is applicable to any data that can be represented as a network.

**Availability and implementation:**

ORCO is an open-source Python package and is publicly available on GitHub at https://github.com/aksimhal/ORC-Omics.

## 1 Introduction

Recent advanced sequencing technologies enable researchers to investigate disease-related biological phenotypes through whole genome-wide landscape scans. As a result, there has been an unprecedented increase in high-resolution biological data such as RNA-seq, DNA-seq, and proteomic sequencing data. Several tools have been developed in the field of bioinformatics to analyze large-scale data, including DESeq2, edgeR, GSEA, and UMAP (Love *et al.* 2014, McInnes *et al.* 2018). However, bioinformatic tools with the capability of assessing interactions via the entire biological system are still lacking. Biological systems can be modeled as undirected networks (or graphs–used interchangeably here) where nodes represent components and edges represent relationships between components. Examples include genomic networks, where nodes represent genes and gene interaction information defines edges, and proteomic networks, where nodes represent proteins and edges represent expression correlation levels.

A key goal of analyzing biological systems represented as networks is understanding critical genes for a given data context as well as the behavior of their local neighborhoods. Many network analysis methods can be applied, such as node connectedness, centrality, and eigenvector centrality (Newman 2018). However, graph analysis tools with the capability to assess the robustness of interactions on a local and global scale are still needed. Additionally, these measures are usually defined for the entire graph or at the node level, not often on the edge level. Graph analysis methods with the capability to assess the robustness of interactions on the edge level within the entire system are still needed. To address this, we have developed a network-based tool, called Ollivier-Ricci curvature-omics (ORCO).

**Figure 1.**
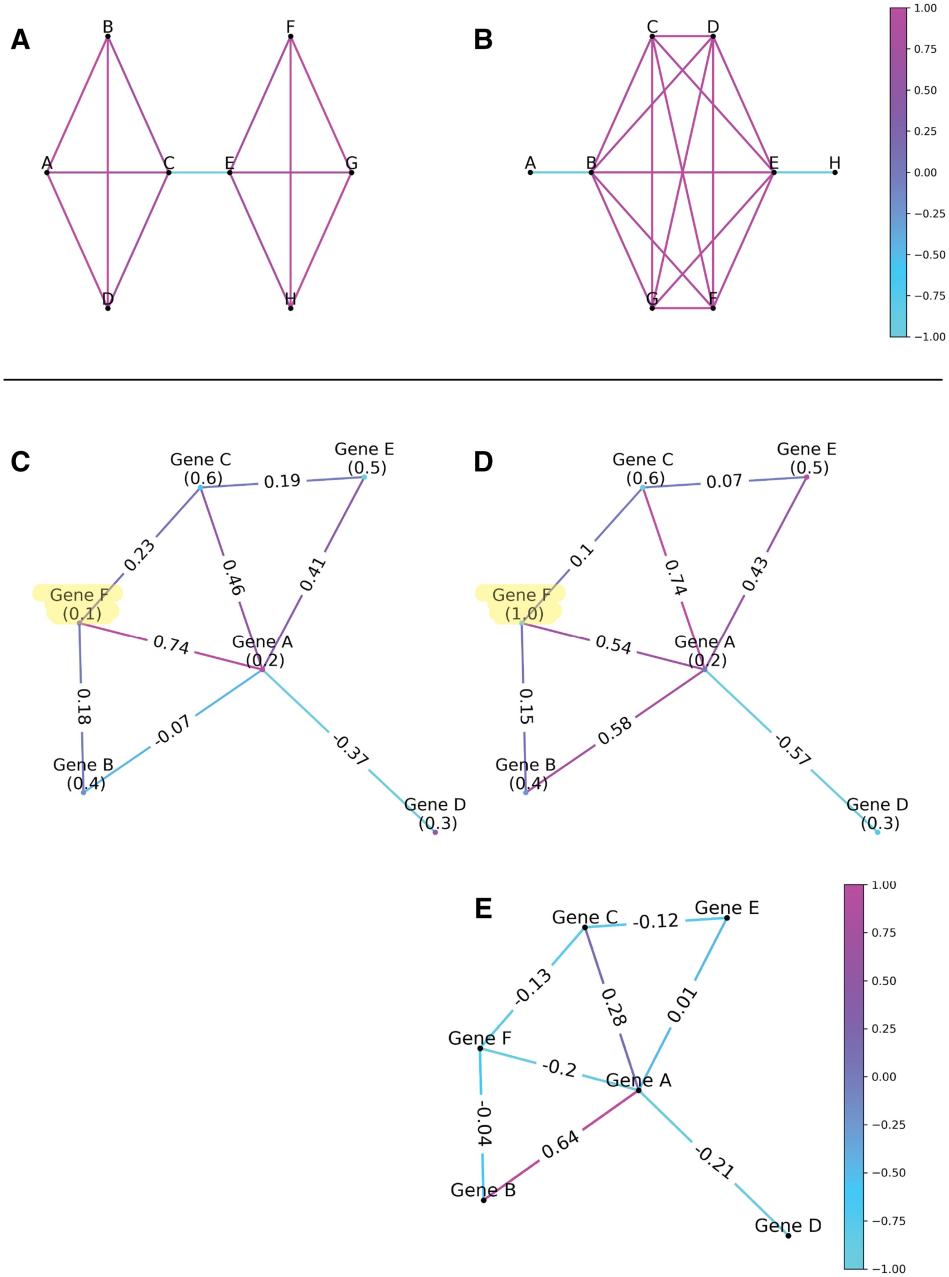
Toy network example demonstrating how ORCO reflects network robustness. (Top row, A and B) Examples illustrating network robustness and fragility. Node weights are uniformly set to 1 and edges are colored by ORC values. Edges between nodes with multiple connections (i.e. triangles) are robust and have positive curvature (purple) while branched edges are fragile and have negative curvature (cyan). (Bottom row) Example demonstrating that changes in curvature reflect changes in network robustness due to changes in node weights associated with functional cooperation. Here, we show how an increase in expression in a single gene, Gene F, can have cascading effects. (C) An example network where nodes are annotated with node weights and edges are annotated with ORC values. (D) The same network but the value of the weight for Gene F has increased 10-fold. Edges are annotated with the corresponding ORC values. (E) The edges are annotated with the resulting change in curvature due to the change in node weight from (C) to (D).

ORCO utilizes Ollivier-Ricci curvature (ORC), an extended notion of Ricci curvature on a Riemannian manifold, defined on a simple, undirected, and connected network (Ollivier 2007). Here, robustness refers to a network’s ability to maintain its structure or function despite perturbations and is indicated by positive curvature. Conversely, fragility denotes the network’s susceptibility to disruption, marked by negative curvature, highlighting sparse or irregular connectivity and critical bottlenecks where perturbations can lead to fragmentation or functional failure. Examples of robust and fragile edges are presented in Fig. 1A and B. In biological networks, robustness often corresponds to conserved, redundant pathways, while fragility identifies vulnerable, disease-relevant regions, providing insights into network stability, disease mechanisms, and therapeutic targets. Examples of robust features include redundancy and feedback loops. For example, redundancy ensures that an electrical power grid continues transmitting even if an individual power station fails. In a protein–protein interaction network, feedback mechanisms may enable a cell to adapt and develop drug resistance. In each case, a lack of robust connectivity between network nodes may indicate a vulnerability in coordinated action. In principle, ORC can be considered to quantify the number of pathways between two nodes. The more pathways exist between a pair of nodes (i.e. positive curvature), the more robust the relationship between the two nodes. Inversely, if removing a single edge in a network makes it impossible for information to pass through (i.e. negative curvature), then that edge is considered fragile. Long-range changes in robustness indicate system-level changes in functional cooperation, which may bear important biological implications. For example, abnormalities in robustness may translate to potential therapeutic targets (Sandhu *et al.* 2015). This metric reflects more realistic biological interactions compared to simple correlations between two nodes. Therefore, the network-based approach could provide unique and unexplored insights into the underlying biology and help identify therapeutic targets and regulatory modules within biological systems, as demonstrated in (Aittokallio and Schwikowski 2006).

ORC has been used in various biomedical studies, including brain connectivity analysis and genomic network analysis in cancer cells (Tannenbaum *et al.* 2015, Farooq *et al.* 2019). In Simhal *et al.* (2020, 2022), ORC was used to elucidate differences in brain connectivity of children with autism spectrum disorders (ASD). It was shown that the information provided by ORC was not simply complimentary to the information displayed by alternative methods but uncovered previously unknown connectivity patterns. In oncology, ORC has been used in a variety of applications. In Simhal *et al.* (2023), ORC was used to identify a novel gene signature for high-risk multiple myeloma. In Elkin *et al.* (2024), a dynamic form of ORC was used to identify therapeutic targets in sarcoma. In Pouryahya *et al.* (2018), Elkin *et al.* (2021), and Murgas *et al.* (2024), ORC was used to identify robust genes while taking into account various types of interaction data. ORC also has been shown to improve the performance of neural networks. In Zhu *et al.* (2023), the authors used ORC to improve the messaging-passing capabilities of their graph neural networks. ORC was also used for community detection (Ni *et al.* 2019). Together, these works demonstrate that the curvature of biological networks can serve as a prognostic biomarker for various disease states, identify previously unknown high-risk patient groups, and rank their unique geometric vulnerabilities.

Previous implementations of ORC require edge weights along with network topology (Ni *et al.* 2019). However, most omics data, such as whole genome sequencing, RNA-sequencing, and flow cytometry consist of node-level values. To address this issue, we present ORC-Omics (ORCO), an open-source Python library explicitly designed for typical bioinformatics network analysis. ORCO intakes node-level data and an undirected network and outputs a network where edge weights represent the robustness between nodes. ORCO provides a quantitative way to measure qualitative notions of “functional cooperation” between nodes in a network. This provides higher-level input regarding system-level function. Additionally, while ORCO is designed for general omic data, it can be used in any context where data is represented as a network.

## 2 Software

The following sections provide a technical overview of the method and input data requirements. We describe the input data using genomic data as an example, but any appropriate data type could be used.

### 2.1 Input data

ORCO requires two input data. The first is a 2D matrix of nonnegative feature data where the columns represent samples and rows represent features (e.g. genes). The second is a 2D adjacency matrix where the dimensions match the number of features in the dataset. This adjacency matrix should be a binary symmetric matrix where a positive value indicates an interaction between features, corresponding to an undirected, simple, and connected network. Other input parameters associated with the ORC formulation may be modified and are described in more detail on the GitHub page. An example input data structure is a comma-separated values (CSV) file with *n* columns where each column represents a sample and *m* rows where each row represents a gene. Depending on the data modality, the value of each cell would be measurements such as gene expression or other omic data.

For genomic data, an adjacency matrix can describe the network topology of genes that are known to physically interact, co-express, or are connected. For general omic data, the adjacency matrix can represent any appropriate type of interaction. Common sources of protein interaction information used to construct a network topology include the Human Protein Reference Database (Peri *et al.* 2004) (HPRD) and the STRING protein–protein interaction network (Mering *et al.* 2003). Hence, our ORCO tool is amenable to various network topologies that can be provided as a symmetric adjacency matrix.

### 2.2 Ollivier-Ricci curvature

A notion of graph distance, defined between every two nodes in the network, is required for computing ORC. The standard hop distance may be used with uniform weights on a given network topology. Alternatively, a more informed weighted hop distance can be computed by considering a random walk on the weighted graph as follows. For each sample, the weights $w_{i}$ for each node i are assigned by mapping measurements to the corresponding node in the graph. The node weights are then used to define transition probabilities from one node to another, which are only nonnegative if there is an edge (i.e. protein interaction) between the corresponding nodes. The transition probability from node i to any neighboring node j, denoted $p_{i,j}$, is computed based on the mass action principle normalized over all neighbors to ensure it is a probability, defined as follows:

(1) pi,j=wj∑k∼iwk, j∼i 0, j ≁ i, 

where k∼i denotes the set of nodes k that have an edge connecting to node i.

Considering that the higher the probability of transitioning from one node to another, the smaller the edge length should be. The edge weights $w_{ij}$ for each edge (*i*, *j*) are transformed as a function of the transition probabilities in each direction as shown in Equation (2):

(2) wi,j=1w∼ij, where w∼ij=pi,j+pj,i2. 

The weighted hop distance between any two nodes i and j, denoted d(i,j), is then the minimum accrued weight over all paths connecting nodes i and j.

Formally, ORC between any two nodes i and j is defined in Equation (3):

(3) κORi,j=1-W1μi,μjdi,j, 

where $W_{1}$ is the Wasserstein distance, also known as the Earth Mover’s distance (EMD), between the probability distributions, $\mu_{i}$ and $\mu_{j}$ associated with nodes i and j, respectively. The probability distribution around a given node (gene), $\mu_{i}$, is defined by the mass action principle, normalized by the net action over all its neighbors, as follows:

(4) μik=rk∑j∼irj, k∼i0, k ≁ i. 

Here *r_k_* denotes the weight of node k (e.g. the RNA-Seq value), and the denominator *d(i, j)* is the weighted shortest path between the two nodes, described above.

The output of this method is a graph with an ORC value for each edge. This output can be analyzed in several ways. Figure 1C–E illustrates an example that shows the cascading effects of overexpression of a single gene throughout the network. ORCO can be installed via Python’s package installer “pip.” The source code, documentation, and examples can be accessed at https://github.com/aksimhal/ORC-Omics. ORCO represents a powerful tool for researchers to analyze genomic data through a network lens.

## 3 Conclusion

ORCO is an open-source tool that provides an easy entry point into Wasserstein-based network analysis. As shown in prior publications, ORC has helped discover novel oncological insights. However, the full potential of ORC is underexplored; there are many biological systems for which ORC has yet to be thoroughly investigated. We hope this tool will assist other researchers with their science.

## Data Availability

Code and notebooks are available at github.com/aksimhal/ORC-Omics.
